# Mechanisms Underlying Auditory Hallucinations—Understanding Perception without Stimulus

**DOI:** 10.3390/brainsci3020642

**Published:** 2013-04-26

**Authors:** Derek K. Tracy, Sukhwinder S. Shergill

**Affiliations:** 1 Cognition, Schizophrenia & Imaging Laboratory, Department of Psychosis Studies, Institute of Psychiatry, King’s College London, London SE5 8AF, UK; 2 Oxleas NHS Foundation Trust, Princess Royal University Hospital, Kent BR6 8NY, UK; 3 National Psychosis Unit, South London and Maudsley NHS Foundation Trust, Bethlem Royal Hospital, Kent BR3 3BX, UK

**Keywords:** auditory hallucinations, neurocognitive, connectivity, fMRI

## Abstract

Auditory verbal hallucinations (AVH) are a common phenomenon, occurring in the “healthy” population as well as in several mental illnesses, most notably schizophrenia. Current thinking supports a spectrum conceptualisation of AVH: several neurocognitive hypotheses of AVH have been proposed, including the “feed-forward” model of failure to provide appropriate information to somatosensory cortices so that stimuli appear unbidden, and an “aberrant memory model” implicating deficient memory processes. Neuroimaging and connectivity studies are in broad agreement with these with a general dysconnectivity between frontotemporal regions involved in language, memory and salience properties. Disappointingly many AVH remain resistant to standard treatments and persist for many years. There is a need to develop novel therapies to augment existing pharmacological and psychological therapies: transcranial magnetic stimulation has emerged as a potential treatment, though more recent clinical data has been less encouraging. Our understanding of AVH remains incomplete though much progress has been made in recent years. We herein provide a broad overview and review of this.

## 1. Introduction: An Overview of Auditory Verbal Hallucinations

### 1.1. Phenomenology and Epidemiology

Auditory verbal hallucinations (AVH) are subjective perceptions of external speech in the absence of external stimuli. They are strongly associated with, and the most common symptom in, schizophrenia—usually intrusive, unintentional, unwanted and distressing—with a one month prevalence of about 70% [1], and are refractory to pharmacological management in about a third of such patients [2]. However AVH are not diagnostic of schizophrenia and occur in other mental illnesses including borderline personality disorder [3], depression, bipolar affective disorders, post-traumatic stress disorder, substance misuse and neuropsychiatric disorders such as dementia, Parkinson’s disease and epilepsy [4].

Phenomenologically they are quite heterogeneous in nature [5]: varying from first to second to third person commentary; from brief utterances of simple sounds or single words to full conversations; consisting of voices (the average is three) from familiar, personal and repeated to the unknown; from passive discussions to issuing commands; and from pleasant or complimentary to—far more commonly—unpleasant and distressing [6]. Traditional teaching has emphasised the location of the percept in external space, though it is not clear that this is always the case, or that sufferers can clearly so delineate them [7]. However AVH generally “sound” like “ordinary” voices with definable characteristics such as pitch, volume and accent. 

Experimental approaches have generally given less salience to the phenomenological qualitative nature of hallucinations [8] as opposed to the more quantitative aspects of occurrence, frequency, level of distress, and response to treatment, particularly as part of a broader illness picture. These latter factors can vary considerably between individuals and, following the frequently episodic nature of mental illnesses, for any given individual.

However, auditory hallucinations are not necessarily pathological and it has been accepted that phenomena such as hypnagogic (occurring just before sleep), hypnopompic (on awakening) and bereavement related hallucinatory experiences are within the spectrum of normal experience. Over recent years there has been a resurgence of interest in the idea of psychotic experiences as lying on a continuum, with non-clinical samples with less intense neutral or positive hallucinatory experiences associated with no functional impact at one end—and hallucinations with greater intensity and negative affect associated with psychotic illness at the other end. In these population based studies, the prevalence of AVH varies significantly, with a recent review [9] of seventeen studies, suggesting a median prevalence of AVH in the healthy population of 13.2% with an interquartile range of 3.1%–19.5%. However the very wide range means that the results of some work, particularly cross-sectional studies and those comprising selective populations, may skew the prevalence results. A recent methodologically robust (*n* = 7075) longitudinal epidemiological study NEMESIS-1 [10] that followed up a stratified sample of 18–64 year olds over three years produced far more conservative figures: 7.8% describing hallucinatory experiences (in any sensory modality) on initial sampling; 2.7% at year 1 follow-up; and 2.0% at year 3 follow-up. Another study from the Netherlands estimated that over 10% of population experience AVH on a recurring basis [11], but the fact that 71% of this cohort did not have purely negative content voices (and 91% reported no disruption to their everyday life from the voices) raises an interesting point about participants’ understanding or concept of what “voices” actually *mean* to the individuals concerned. Distress about AVH has been shown to be proportionate to the *belief* in the voice(s), rather than what it/they say, and the lack of control over their occurrence [12]. Other factors need to be considered when contextualising AVH frequency. Stress and tiredness appear to be common precipitating factors for AVH, whether with background mental illness or not [13]. There are significant gender [14] ethnic and cultural variations [15] in such phenomena, likely reflecting differences of explanation of inner psychic phenomena, acceptability and stigma that will further bias reporting [9]. In summary, the prevalence of AVH in general population samples requires caution in interpretation and awareness that many factors influence the data—including study methods and representativeness of samples, perceived stigma, ethnographic and individual constructs of what “voices” are or mean, and variation in phenomenological strength or severity (and study cut-off threshold) of such symptoms.

### 1.2. AVH as a Continuum Phenomenon?

The significance of these findings in the so-called “healthy” population, and whether or not it fits with a broader model of a psychosis spectrum [11] or a continuum of “normal” experiences [16,17] remains contentious [8]. Sommer *et al.* [11] demonstrated a marked increase on all sub-clusters of both the Schizotypal Personality Questionnaire (SPQ) and the Peters Delusion Inventory (PDI) in 103 otherwise healthy individuals with AVH (mean period of 29 years) compared to 60 matched controls. However the participants with AVH did not have significant delusions, disorganised or negative clusters of symptoms, and their scores on the SPQ and PDI were insufficient to meet diagnostic criteria for a schizotypal disorder or personality disorder, though the raised scores were not due to sub-group of high scorers but reflected a good across-group change. Nevertheless factors such as age of onset (later in schizophrenia), frequency, duration, (negative) emotional content, experience of (lack of) control, and subsequent dysfunction would appear to delineate schizophrenia from non-schizophrenia AVH [8,18]. One other potentially significant difference noted by Johns *et al.* [13] is the tendency for patients with schizophrenia not to challenge the origin of such voices. This fits with a broader illness cognitive bias, more typically malevolent misattribution, and reduced reappraisal [19] that might lead to distress, dysfunction and ultimately both to presentation to professionals and serve as a factor in maintaining AVHs. A more recent study examining social cognition in relatives and patients suggest that contextual flexibility may hold the key to differentiating those at risk of psychosis from those who become psychotic [20]. 

Healthy individuals with AVH have greater rates of mental illness in biological relatives and genetic links have been demonstrated between psychotic and neurotic disorders [21,22,23]: a genetic loading for AVH as well as shared environmental issues and psychosocial stresses are likely to be relevant predisposing, precipitating and perpetuating factors. The question of whether hallucinations or delusions “come first” in psychotic illnesses remains an issue of debate. The aberrant salience hypothesis [24] posits that anomalous interpretations could account for both phenomena, with delusions a higher-level cognitive attempt to explain abnormal experiences, and hallucinations the erroneous salience of an internal representation of a percept or memory. This conceptualisation also potentially affords an explanation to the continuum question insofar as it permits a gradated spectrum of hallucinatory experience from an internal monologue or inner voice through increasingly alien experiences of “voices” that are more “external”. Whilst salience dysregulation does not necessarily demand that AVH precede delusions, this has often been argued, with delusions seen as secondary phenomena explaining the voices [25], though there are alternative cognitive models that allow for the independent development of delusions [26,27,28,29]. The previously mentioned NEMESIS-1 study [10] also showed that AVH and delusions in the general population cluster together more often than predicted by chance. In this work the presence of AVH increased the risk for future development of other first rank symptoms of schizophrenia, and the occurrence of both AVH and delusions was more strongly associated with greater familial loading and risk exposure than either symptom alone. The authors hypothesise that a critical step in the development of psychotic illnesses occurs when sub-threshold symptoms in each domain combine in an exacerbatory manner, though the epidemiological nature of the study did not allow attribution of causality between them. 

Mental illnesses are increasingly considered as dimensional and spectrum disorders of varying overlap and severity, and psychotic and affective symptoms commonly, though not inevitably, co-occur [30,31,32], although categorical diagnostic systems mean they are often treated, studied and conceptualised as separate [33]. The majority of sufferers of schizophrenia, including those with “non-affective” illnesses, have symptoms of depression and anxiety in their illness history [34], and longitudinal work [35] has shown that negative cognitions maintain paranoia, and an individual’s affect may be a perpetuating factor in AVH persistence in schizophrenia. 

In general the rate of psychotic symptoms in affective disorders has been less studied than affective symptoms in psychotic disorders [36]. A cross-sectional study of over a thousand patients at an American urban primary care practice [37] found psychotic symptoms, most commonly AVH, in 20.9%: such patients were significantly more likely than those without psychotic symptoms to have a major depressive disorder (42.4% *vs.* 12.6%), panic disorder (24.8% *vs.* 4.0%), generalised anxiety disorder (38.6% *vs.* 8.4%) and alcohol misuse (12.9% *vs.* 5%), as well as worse socioeconomic circumstances. Data from the Early Developmental Stages of Psychopathology (EDSP) study [36] has demonstrated that approximately a quarter of adolescents and young adults in a representative community sample (*n* = 3021) of those with depressive and/or anxiety disorders (and no previous psychotic illness) displayed at least one psychotic symptom. The large and influential STAR*D study of outcomes in depression identified wide ethnic variation in the prevalence of AVH in highly representative US population study of participants with a major depressive disorder [38]: 2.5% of white participants (*n* = 49/1928), 11.3% of black participants (*n* = 45/398) and 6.3% of latino participants (*n* = 17/270). A significant association was found in all groups between the presence of AVH and comorbid PTSD and panic disorder. A recurring finding across most of these studies was not just the common occurrence of symptom overlap between affective and psychotic disorders, but also that such coexistence was associated with worse outcomes.

## 2. The Neurocognitive Models

Several possible neurocognitive models of AVH have been proposed, with the two most consistently supported noted below. It remains unclear if a single model will emerge as “correct”, or if varying pathologies may account for these symptoms in different people, particularly between various clinical and non-clinical groups. 

### 2.1. The Feed-Forward Model

An aberrant “feed-forward” mechanism [39] has remained an influential neurocognitive model underlying passivity experiences in psychosis, such as delusions of control and AVH, for over two decades. Refined into a Neurocognitive Action Self-monitoring System (NASS) [40] this model proposes that all motor activity involves sending an efferent corollary discharge of planned actions to the relevant sensory cortex, allowing an evolutionarily sensible mechanism for prediction of motor actions with sensory feedback and refinement of the planned act. Comparison and matching of predicted and received sensory input attenuates the signal, with less conscious attention paid to the act, whilst variation between prediction and outcome raises awareness levels and evaluation of this process. 

Pathological disjoint between the prediction and outcome self-monitoring leads to greater parietal sensory cortex activation [41,42], as would happen from a genuinely external stimulus. This may result in the experience being perceived as external in origin, sometimes referred to as autonoetic agnosia, or an inability to recognise self-generated events [43]. Earlier work by our group testing this [44] involved participants (both with schizophrenia and healthy controls) having to replicate a force applied by a motor to their left index finger, either through direct application of pressure via their right index finger, or indirectly via a joystick. Fitting with the NASS sensory attenuation model all participants consistently applied greater direct force, and were less accurate, via direct self-application of the force —where sensory expectation of their finger press reduced the sensation—than indirectly via a joystick. Individuals with schizophrenia were *more accurate* than healthy controls: this seeming paradox in a sensory dysfunction model is potentially due to their failure to send an appropriate sensory efferent that would attenuate the signal. A meta-analysis by Waters *et al.* [45] of 32 studies demonstrated significantly reduced self-monitoring in patients with schizophrenia compared to healthy controls, and within the schizophrenia group this was more pronounced in those with AVH compared to those without. Most recently our lab has undertaken a neuroimaging study [46] comparing patients with schizophrenia to healthy controls undertaking either a cued or spontaneous motor task. The spontaneous task elicited significantly greater activation in the patient group’s left inferior parietal lobe (IPL), which is part of the secondary somatosensory cortex, than that of the controls or of the cued task. This is in keeping with the principle that predictable actions, which the cued task would better model, produced less activation of the sensory cortex [47]. Supporting this, a follow-up scan of the patient group at six to eight weeks, by which time their positive symptom score (as measure by the PANSS scale) had reduced, showed left IPL activation returning to normal.

Seal *et al.* [48] applied the NASS model to inner speech, the subjective experience of talking to oneself, which is accompanied by the motor process of laryngeal sub-vocalisations. They proposed that deficient self-monitoring was a key deficit, with arrival of inner speech sensory input, but an absence of an appropriate prediction of this, leading to such speech appearing *unintended*. Our group had previously shown that patients with schizophrenia and AVH demonstrated reduced activation, compared to healthy controls, in regions implicated in verbal self-monitoring whilst generating inner speech [49]. The generation of auditory verbal imagery, that is imagining the speech of others, used a similar speech network, but the postulated increased demands of this resulted in even greater activation of self-monitoring regions [50]. 

**Figure 1:**
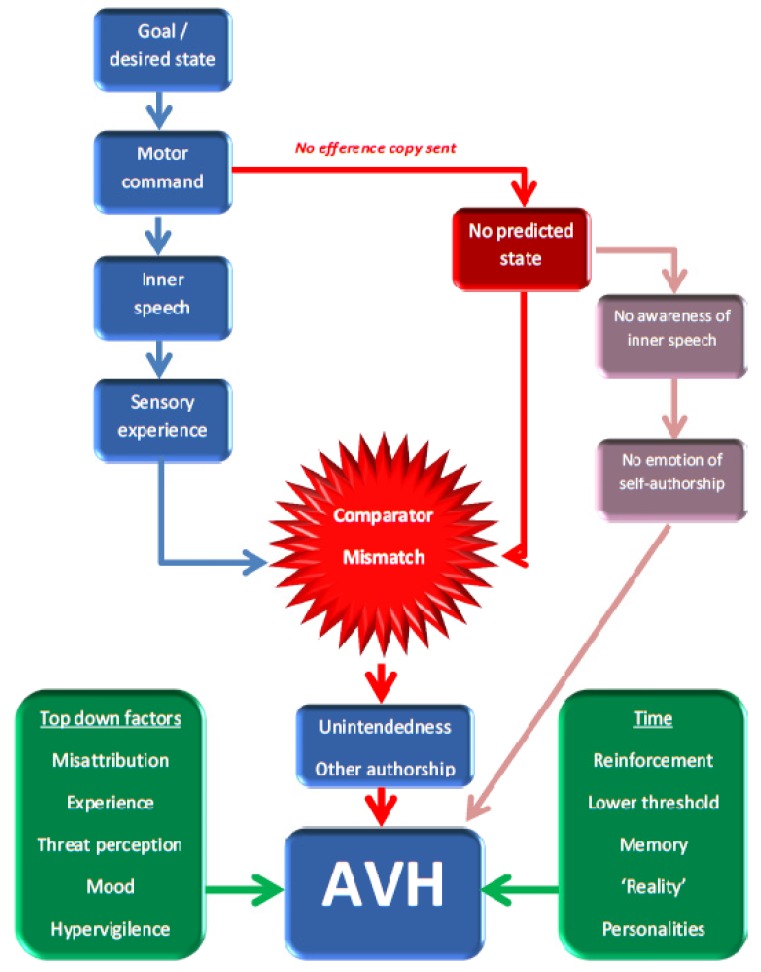
The feed-forward model of auditory verbal hallucinations (AVH). Failure to send an efference copy of intended inner speech leads to a comparator mismatch between sensory experience and expectation, leading potentially either to a sense of “unintendedness” (Seal’s model) or “other authorship” (Jones’ and Fernyhough’s model). Top-down factors contribute to a propensity to further misattribute this perception as alien, and over time lowered thresholds to accept such as external may arise, with “hard-wiring” of the voices. Signal Detection Theory gives greater weight to the role of top down factors, but is compatible with the other aspects.

Other pathological processes in psychosis, including deficient top-down factors [51] such as episodic memory and past experience, attributional biases, mood, and expectations helped confirm the *misperception*. A more recent modification by Jones and Fernyhough [52] suggests that the failure of a predicted state results in a lack of agency, with the dysfunctional mismatch of the predictive state and actual sensory experience that led to a sense of external authorship of the “voice”, negating the need of “intendedness”. A further variation of these is the Signal Detection Theory (SDT): under this model the perceptions can be normal (though there *could* also be aberrant signalling), but there is ambiguity in all signal recognition. The model posits that it is pathological misattribution and source-monitoring that leads to the externalising of the perception, potentially exacerbated by concomitant hypervigilence and perception of threat [53]. Social development studies have shown the ability to trust and mentalisation of the actions of others increases with age [54], but that patients with psychotic illnesses show impairment in them and reduced ability to modify their behaviour in these domains despite feedback [20]. The SDT model is compatible with earlier ones, and has the attraction that—for schizophrenia at least—it links with established cognitive biases and other psychopathology such as delusions, as well as readily offering an explanation of the efficacy of antipsychotic medication for AVH. In all these models time altered expectations may result in a propensity to accept them as real, personalised, definable, and different voices [55]. Figure 1 gives an overview of these models.

### 2.2. The Aberrant Memory Model

**Figure 2:**
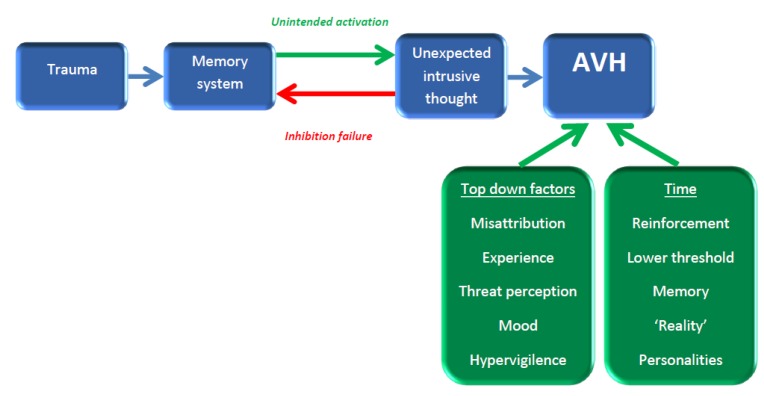
The aberrant memory of AVH. Traumatic memories are prone to be inappropriately, intrusively and unexpectedly brought to consciousness through a combination of unintended, non-contextualised activation and higher inhibitory failure. Their unexpected contextualised nature and, as with the feed-forward model, the influences of top down and temporal factors lead to the perception of AVH.

A second model postulates that AVH result from aberrant memory activation and monitoring, particularly from past traumatic experiences, potentially due to both a failure in inhibition of recall and unintended memory activation. The link between trauma, particularly early childhood and sexual abuse, and the later development of AVH appears to be a solid one [14,56,57]. Failure of inhibition may generate intrusive thoughts, which are increased in schizophrenia, despite also occurring in other mental illnesses and even in healthy individuals. Waters *et al.* [58] suggest that the unintended and out-of-context memory activation is due to dysfunctional prefrontal inhibition. The content of the memory, arising out of context, leads to the sensation of “otherness” and external authorship that results in AVH. Figure 2 illustrates this model.

Working memory performance does not seem to be affected by AVH, though fMRI data does show differential activation of related cortical areas between hallucinators and non-hallucinators in schizophrenia [59] and the behavioural data is conceivably insensitive or testing different aspects to any AVH pathology. However episodic memory deficits in patients with schizophrenia are well established, and memory processing involves at least similar brain regions to those activated in AVH such as the temporal cortices and hippocampus (as discussed in the next section), but it has only been more recently that efforts to delineate the two distinct neural processes of encoding the relevant data, and also the spatio-temporal context in which it occurred, have been made.

Work by Brebion *et al.* [60,61] explored these differential memory components—and correlated against symptomatology—by looking at a dual aspect memory recall task that involved both remembering word lists (the information) and also assigning them to the correct list from which they were presented (the temporal context). List misattribution had a greater error rate in patients than healthy controls, and within the patient group it was higher in those with AVH (though not in those with delusions)—in the latter case independent of verbal recall efficiency. Interestingly errors were inversely correlated with negative symptoms: this unexpected finding has no clear explanation though the authors postulate that such individuals might have diminished impulsivity.

This model is also in concordance with data showing that “normal” sensory experiences are not passively stored in memory, but are affected by top-down processes such as expectation, to form an idiosyncratic and personalised subjective reality perception [62]. Waters *et al.* [55] note that a pathological version of this could lead to “hard wiring” with time of sensory and reality distortions.

### 2.3. Critiquing the Neurocognitive Models

Current neurocognitive models of AVH are incomplete. Although AVH occur outside of psychosis, they are the most common symptom of schizophrenia but problematically the postulated models do not clearly fit with the predominant psychopharmacologically motivated theory of schizophrenia, the dopaminergic hypothesis, with the neurotransmitter dopamine having a key role as the “wind of the psychotic fire” [63]. This provides an optimal model for positive and negative symptoms of schizophrenia, rather than AVH, and brain regions implicated in this dysfunctional model (see next section) do not contain significant levels of dopaminergic neurons [64]. The dopaminergic hypothesis is itself contentious, although it explains the efficacy of dopamine blocking medications in reducing AVH for many patients [65]. Nevertheless the overactive mesolimbic reward learning system could potentially link with attributional salience and top down factors [66]. This also accords with the strength of belief in the voice (a salience/delusional component), a key factor correlating with the level of distress, and a delineating factor for less/non-distressing AVH in the healthy population. However should this be the case it is unclear if both types of deficit—deficient self-monitoring and higher cognitive appraisal—are part of the same pathological process, and if effective dopaminergic blocking medication alters the latter misattribution, does the pathological feed-forward aspect persist, and if so why are such abnormal speech processes still not detected? No authoritative cause for any primary deficit in the auditory cortices has yet been proposed. Finally not all studies on AVH have included non-hallucinating patient populations, which raises a question on the specificity of any detected abnormalities [67].

Regarding the feed-forward model, the ability to determine internally versus externally generated motor actions, including allowing any necessary action modification or refinement for the former, would seem to be an evolutionary logical advance. However it is less clear that this can be applied to inner speech where there is no obvious need to be able to differentiate “own” from “other” thoughts, nor that planned models would be needed to allow for refinement or adjustment as external motor actions might [52]. The NASS model is not unique to AVH, but can be applied to other psychotic pathology, indicating that aberrant feed-forward is unlikely to be sufficient for AVH to occur [55]. Gallagher [68] also highlights the issue of “unbidden” thoughts that “spring to mind”, which are a universal experience, but are never ordinarily given external authorship despite their unexpected occurrence, though it remains possible (and likely) that although unanticipated they had subconscious corollary efferents generated. 

Furthermore if simply misattributed inner speech the question arises of why AVH never have the same acoustic properties as the hallucinator’s own inner voice, but take on the attributes of another’s (or others’) speech patterns in what can be a stable fashion. One partial explanation involves the concept of prosody, which incorporates non-lexical aspects of speech [69], such as intonation, emotion and accent, and individuals with schizophrenia have been shown to have deficits of prosodic comprehension [70]. It is conceivable, though speculative, that this is one of the top-down deficits that contribute to the external attribution of AVH, though it doesn’t account for the fact that “normal” inner speech that co-occurs with AVH does not take on such qualities; it does not answer the question of why AVH are usually experienced as unpleasant; and does not really account for how consistently different—including difference in gender—AVH can be from the sufferer’s own inner voice [71]. However in support of a prosodic component work by Allen *et al.* has shown that misattribution of self-generated speech was more likely when it had a higher emotional content [72].

Critiquing the “memory hypothesis”, clearly trauma is neither necessary nor sufficient for the development of AVH. Whilst it has repeatedly been demonstrated that for individuals who suffer significant trauma the voices can repeat utterances or memories of the abuse, this would appear to be a minority experience. It also does not fit with the frequent finding that voices can “evolve” conversations over time [7], can comment on current happenings, can involve “simple” noises and music, and sometimes be engaged with and questioned. The relationship between problematic spatio-temporal memory encoding and AVH might just represent a more general phenomenon of dysconnectivity between regions used for parallel and related processes of memory and speech. Overall there has not been strong conceptual, phenomenological or empirical support for this model. 

## 3. Imaging Data

### 3.1. Initial Imaging Data and Structural Scans

Neuroimaging studies have been carried out on AVH for over 25 years, the majority of them on patients with schizophrenia. Early work used positron emission tomography (PET) and single photon emission computed tomography (SPECT) methods—which, through their use of ionising radiation, inherently limited participant numbers—and demonstrated a network of activations including subcortical regions such as the thalamus and striatum; limbic regions, especially the hippocampus; paralimbic regions such as the orbitofrontal cortex and the parahippocampal and cingulate gyri; and cortical auditory areas including the left superior temporal gyrus [73,74,75]. Structural MRI studies have shown reduced grey matter volumes in implicated speech regions [76]: a meta-analysis by Modinos *et al.* [77] of nine structural studies on patients with AVH demonstrated volume loss in the superior temporal gyri bilaterally that was correlated with symptom severity. Recent diffusion Tensor imaging studies have implicated lack of white matter integrity in key fronto-temporal and parietal tracts in psychosis with a more complex picture of increased integrity in local temporo-parietal loops [78]. However the very nature of such data means it is difficult to make more than implicit support for neurocognitive models of AVH.

### 3.2. fMRI: Building up a Network of Involved Regions

SPECT and PET have been largely superseded by functional magnetic resonance imaging (fMRI). There is variation between such studies that, at least in part, represents different experimental paradigms and participant variables, including the type of control group used and numbers of participants. However following the discussion on neurocognitive models, neuroimaging differences might also occur from fundamentally varying intra-individual pathological processes.

Nevertheless a reasonably robust pattern of implicated regions has emerged including the left inferior frontal gyrus [49,79,80,81,82,83]; the right inferior frontal gyrus [79,80,83,84]; the parahippocampal gyrus and hippocampus [49,79,84]; the middle and superior temporal gyri [49,79,80,82,84,85]; and the thalamus [49,80,85]. 

This network is in concordance with neurocognitive models, implicating fronto-temporal regions associated with speech generation and perception and medial temporal regions involved in verbal memory. A coordinate based meta-analysis by Jardri *et al.* [86] of both PET and fMRI studies calculated that the regions most consistently associated with AVH were: Broca’s area; the anterior insula; the precentral gyrus; the frontal operculum; the middle and superior temporal gyri; the inferior parietal lobule; and the hippocampus and parahippocampal region. 

### 3.3. State and Trait Analysis

Kühn and Gallinat [87] recently undertook a meta-analysis of neuroimaging data on *state* and *trait* aspects of AVH in schizophrenia: that is those areas whose activation varied within-subjects (hallucinators) depending upon the presence or absence of AVH (state); and those areas whose activation varied between groups of hallucinators and non-hallucinators (trait). Their analysis showed state studies associated with bilateral inferior frontal and postcentral gyri, and left parietal operculum activation; whilst trait studies showed hallucinators had attenuated activation in the left superior and middle temporal gyri and the left premotor cortex. The nature of comparing such studies means that the direction of difference is difficult to determine in the trait data: apparent hypoactivation in the AVH symptomatic group could be caused by either lower *or* higher background tonic activity in the relevant regions as fMRI analysis will only measure within-subject changes in these areas between test paradigms. However the authors postulate that these results accord with general auditory cortex changes predisposing to the trait of AVH and specific speech production regions, such as Broca’s being implicated in their pathological state occurrence. Allen *et al.* [88] noted an inherent difficulty of state studies wherein the precise role of regions of activation in an AVH physiological pathway can only be inferred, and posited that trait studies are better suited to elucidate this.

### 3.4. Non-Schizophrenia Populations

Linden *et al.* [89] undertook the first fMRI study of a non-clinical AVH population, comparing hallucinations with auditory imagery in seven participants with no history of psychiatric or neurological disorders, or of any illicit drug use. Frontotemporal language areas were activated by both tasks, but critically, the timing of the supplementary motor area (SMA) activation differed, preceding auditory areas in the imagery task but occurring simultaneously with it during AVH. The SMA has been implicated in movement planning and the parallel (rather than preceding) activation would fit with a failure of a feed-forward process.

A larger study, with an active control group, by Dierderen *et al.* [90] compared 21 non-psychotic participants with AVH but no psychiatric illness to the same number of psychotic participants with AVH matched for age, gender and quantitative markers of their hallucinations (such as frequency and duration). A similar pattern was seen between the two groups, with activation in a fronto-temporal speech network identified in other studies including the bilateral inferior frontal gyri, insula, superior temporal gyri and postcentral gyri, left precentral gyrus, inferior parietal lobule, superior temporal pole and right cerebellum. No statistically significant differences were elicited between the two groups, leading the authors to propose the same cortical network underlies AVH activation in both groups. 

### 3.5. Challenges in Functional Imaging Studies

AVH occurrences are not predictable, and “capturing” them in a scanner is not without problems. Studies have alternatively tried to compare within-individual data looking for differences when AVH occur, and also trying to delineate cerebral activation between “hallucinators” and non-hallucinators (both those with schizophrenia and healthy controls). Various techniques to “provoke” relevant activation have been utilised, including generation of inner speech and listening to speech; as well as alternative methods to “capture” AVH including button presses and random brain sampling. However identification of speech regions does not tell us why they become dysfunctional in the first place, nor why their activation leads to misperception of internally generated stimuli as external in origin [91]. Undoubtedly there has been insufficient work on the neurobiology and neuroimaging of AVH in “healthy” populations. Indeed there has been no work to date exploring neuroimaged similarities and differences with other patient groups, such as bipolar affective disorders or psychotic depression.

Finally the actual methodology involved in fMRI analysis is prone to serious potential bias. Many studies have had small sample sizes and issues persist about the confounding effects of psychotropic medication and non-specific illness related cognitive effects such as more generalised language dysfunction and thought disorder [88]. No functional imaging study has identified a causal site of brain pathology in schizophrenia (or any other functional mental illness) [92]. The issue of “voxel shopping” whereby multiple testing and model modifications can produce spurious results is a real concern to the reader in interpreting such data, particularly if such analysis has not been disclosed or the methods section is insufficiently clear. A recent paper by Vul and Pashler [93] nicely highlights this problem, which they identify as a form of publication bias in neuroimaging: the authors surveyed the literature for papers reporting high degrees of correlation between social behaviour and focal brain activation (not specifically related to schizophrenia per se), and found the majority contained a circular reasoning insofar as the loci identified were chosen because of the correlation itself. fMRI data sets invariably contain many voxels of activation, depending upon the threshold set, with varying levels of “noise”, and those that pass a given filter threshold are disproportionately likely to have had greater noise interference, though this cannot be corrected for as the degree is unknown. Interestingly correcting for multiple comparisons in fMRI data sets can increase the problem as the process of raising the threshold to a more conservative level furthers the overestimation of the signal strength. Kapur *et al.* [94] note the broader issue of “significance chasing” and “approximate replications” with biological tests in psychiatry: large amounts of publications report statistically significant but underpowered findings with small or moderate effect sizes of limited utility or real value, only to be seemingly furthered by a superficially novel (and equally underpowered) replication that adds to the problem of publication bias. The large international “1000 Connectomes” project is lauded as an example of a forward thinking solution to this in neuroimaging, with 35 laboratories in ten countries collaborating to provide enormous potential power to future studies. 

## 4. Dysconnectivity as the Common Mechanism? Joining the Cognitive Model and the Imaging

### 4.1. Normal Connectivity: Intrinsic and Extrinsic Networks

Data from healthy volunteers demonstrates regions of locally rich high-clustering interconnections in modular arrangements in the sensory cortices that interface through integrative attentional and salience hubs of massive intra-regional connectivity—sometimes referred to as fat-tailed degree distribution or rich club hubs—to higher level cognitive functions [95,96,97]. Both task based and “resting”/non-task based methodological paradigms have been employed to explore these large-scale networks, with various analytical modelling methods such as dynamic causal modelling, independent component analysis, graph theory, psycho-physiological interaction and clustering. The technique of Functional Connectivity is an application of fMRI analysis—known as fcMRI—to computationally model chronoarchitectural connections between identified regions of activation, so-called “connectomics” or the “connectome”: this can explore both modular networked hub centres and more global hierarchical brain connections, and examine data from a voxel to a region-of-interest level [98]. 

Most research supports the functional organisation of normal brain activity into two anti-correlated large competitive networks of intrinsic and extrinsic activity [99]. The so-called Default Mode Network (DMN) or Task-Negative Network is a functionally dominant non-goal orientated background (or intrinsic) resting state associated with, and showing increased activation during, inner speech and introspective contemplative thinking [100], and deactivating during tasks requiring higher levels of cognition or goal directed thinking. It accounts for about a third of waking time [101] and includes regions of the medial prefrontal cortex, lateral and medial temporal lobes, lateral parietal cortices and the posterior cingulate cortex [102]. The DMN can be switched by sensory information to an attention demanding goal-driven extrinsic mode through an attentional network that includes the dorsolateral prefrontal cortex, frontal eye fields, inferior precentral sulcus, middle temporal motion complex and superior parietal lobule [103]. A recent review of fcMRI of normal brain functioning by Sepulcre *et al.* [104] noted the advancement of two critical interface hubs: firstly the temporo-parietal junction as a pivotal component and mediator of the DMN and attentional and control processes. Secondly the posterior cingulate cortex and precuneus appear to act as an integrative locus for the convergence of inner speech and cognitive control systems through connections with the hippocampus, medial temporal lobe and ventromedial prefrontal cortex. 

Doucet *et al.* [105] propose that the intrinsic network can be subdivided into three modules subserving spontaneous thought generation, maintenance and manipulation of information, and cognitive control and switching activity; and further that the extrinsic network has two major modules concerning auditory and primary somatosensory information, and visual areas respectively. A large recent study by this group [99] explored haemodynamic low frequency oscillations and temporal connections, utilising inner speech and visual imagery, in over three hundred healthy volunteers. Free-flowing thinking in images and words led to a reduction in temporal connectivity both between different intrinsic regions and particularly with extrinsic goal driven regions. The lateral parietal and frontal regions, most implicated in maintaining and manipulating inner thoughts, were most associated with intrinsic activity. 

### 4.2. Connectivity Analysis in Schizophrenia

The very word schizophrenia arises from a 19th century conceptualisation of a schism of the mind (“phren”), and the current Japanese term for the illness—Togo Shitcho Sho—translates as integration-dysregulation syndrome [106]. This illness has served as a paradigmatic model for moving beyond purely cellular and neurochemical brain changes to exploring functional dysconnectivity. Several recent reviews have broadly explored the existing literature and evidence for dysconnectivity in schizophrenia [107,108,109,110,111]. 

Overall there is evidence for widespread reductions in connectivity, with prefrontal cortical dysconnectivity being the most replicated finding, particularly with alterations to fronto-temporal connectivity. Current techniques cannot determine if such a reduction is due to global pathology or a single (or few) node(s) with widespread, and locally variable, effects. Whilst most work has found reduced connectivity, hyperconnectivity has also been identified, as has variation within patient groups, including differences related to varying symptoms. More broadly a general principle of *dys*connectivity arises, including potential plastic compensatory changes. Disconnects between the DMN and the extrinsic goal orientation state, potentially via a dysfunctional salience network involving the anterior cingulate cortex and insula have been proposed as a mechanism [112], and increased activation of the former has been shown to associated with clinical improvement and reduction in of AVH in schizophrenia [113]. There are data supporting both independent background state deficits and superimposed local-circuit trait dependent changes, though it has proven complex and technically difficult thus far to confidently delineate these and there is a significant lack of longitudinal data. Emerging data support a link between functional and anatomical changes in schizophrenia, though the strength and nature of these has yet to be elucidated. 

### 4.3. Dysconnectivity and AVH

Earlier work by our lab [114] explored temporal brain changes by getting participants (hallucinators) to initiate a button press on commencement of a hallucination, and to release it when the voice finished. Each AVH lasted on average 16 s (with a range of 3–42 s): analysis of this data demonstrated activation of the left inferior frontal and right middle temporal gyri between six and nine seconds before individuals indicated the occurrence of AVH, whilst the temporal gyri bilaterally and the left insular activation were concomitant with it. However the sample size was small (*n* = 6) and more recent work has undertaken similar, though larger studies [115,116,117]. A replication of this study with larger numbers [116] included both hallucinators (*n* = 11) and patients (*n* = 10) with similar diagnoses of schizophrenia but who did not hallucinate. Again the left inferior frontal gyrus was found to activate, significantly more greatly than its contralateral right sided homologue *before* AVH perception: bitemporal activation was also demonstrated, greater in the right posterior region. The correlation between the left inferior frontal gyrus and the right temporal region was greater in the hallucinator group, and interestingly this group also showed a delayed (after AVH perception) activation in the left temporal homologue. These data make conceptual sense insofar as they would fit with regions involved in auditory verbal imagery, inner speech generation and heightened auditory attention [116,118,119] preceding perceptual experiences, and are congruent with the feed-forward model: the delayed left temporal activation is conceivably linked with aberrant efferent motor planning signalling. A more recent study compared 24 hallucinators with psychotic illnesses, who indicated the presence of AVH through balloon squeezing, to 15 healthy controls with matched balloon squeezes. A “selective averaging” method was used to evaluate activation preceding the onset of AVH. As well as obtaining expected bilateral fronto-temporal activation during AVH (right hemisphere greater than left) there was significant deactivation of the left parahippocampal gyrus [115]. The parahippocampus has a role in memory recall, with connections to the association cortices including language regions and the hippocampus. It also has dopaminergic innervation, and the authors postulated that pathological hyperdopaminergia in schizophrenia might alter efficient neural processing in these regions, and contribute to the hallmark non-recognition of AVH. 

Vercammen *et al.* [117] took the temporo-parietal junctions (TPJ) bilaterally as seed regions to explore resting functional connectivity with a priori defined regions of interest in the postulated inner speech and AVH network in 27 patients with schizophrenia and a similar number of healthy controls. Their results showed abnormal connectivity in the left TPJ: with the right sided homologue of Broca’s area in the patient group compared to healthy controls; and with a further correlation between symptom severity and decreased connectivity between this seed area and the bilateral anterior cingulate and amygdala, areas the authors note are linked with attribution of agency, self-referential and attentional processing. The reduced connection with the right homologue of Broca’s is interesting, as speech generation is classically considered a left hemispheric phenomenon (in right handed individuals), though the right side has been shown to have greater prosodic involvement.

The largest such study to date, by Hoffman *et al.* [120], utilised fcMRI—seeded from a bilateral Wernicke’s region—in 32 patients with schizophrenia reporting AVH, 24 patients without AVH, and 23 healthy controls. Seeded functional connectivity in the left IFG was significantly greater for the hallucinating patients than the non-hallucinating comparators, but not compared with healthy controls. However seeded functional connectivity was significantly greater for the combined patient group, compared to the healthy controls, in a subcortical region including the thalamus, midbrain and putamen: the latter showed significantly greater functional connectivity (relative to a secondary left IFG seed region) in hallucinators compared to non-hallucinators, implicating a key role for the putamen. The inclusion of the non-hallucinating patient group was novel, and authors posit that these data are consistent with a hyperconnected level of functional coordination being intrinsic to a corticostriatal loop or network leading to episodic co-activation as a hallucinogenic causal factor.

Overall these studies suggest a common theme of fronto-temporal dysconnectivity, which is the most consistently replicated finding in fcMRI work, and fits with AVH neurocognitive models, involving as it does key language production and comprehension centres. Discrepancies between trials are likely due in part to the confounders facing all such studies: participant numbers (and use and type of controls), use or absence of behaviour tasks, method of data analysis and so forth. 

### 4.4. Transcranial Magnetic Stimulation: Altering Connectivity?

Repetitive Transcranial Magnetic Stimulation (rTMS) is a relatively new, non-intrusive and painless neuromodulatory technique that utilises Faraday’s Law of induction wherein an alternating (repetitive) magnetic field applied to the head induces electrical depolarisation of underlying neurons, with the rate of the magnetic coil’s activation dampening (<1 Hz) or exciting (>10 Hz) directly underlying cortical activity [121]. Given both the limitations of current treatments and known or postulated pathophysiology in AVH, there has been much interest in whether or not the dysfunctional network can be re-regulated via rTMS. Work by our lab on healthy volunteers demonstrated not just anticipated localised attenuation of the underlying right temporoparietal cortex by slow rTMS, but alterations to connected frontal regions, including contralaterally, and the unexpected finding of an apparently plastic increase in the contralateral temporoparietal homologue [122]. A follow-on study [123] showed that this rTMS paradigm strengthened connectivity between the right temporoparietal cortex and the dorsolateral prefrontal cortex and the angular gyrus. These data highlight how rTMS effects might arise not just from direct effects to the underlying cortex, but through alteration of connected networks. 

To date there has been disagreement in the literature surrounding its efficacy, with some work showing positive results, but others not replicating this. One core problem is a huge variability in trial methodologies, including participant eligibility, symptom measurement, and a lack of consensus about many aspects of rTMS application. The TMS coil can be sited in a number of ways—via electroencephalogram marker points, by localisation of neurophysiological markers in the motor cortex and through fMRI guidance—each one with differing advantages and problems, and either active or sham controls can be used. The optimum duration of a single session (typically around 15 min), the number of sessions (usually daily for a fortnight) and the need or utility of follow-up sessions after the initial treatment remain unanswered questions. 

The most recent meta-analysis [124] from 17 RCTs is in agreement with the four previous meta-analyses [125,126,127,128] in supporting the efficacy of rTMS in treating AVH. However the inclusion of some more recent, larger, studies (including some currently being written up for publication) with negative findings has led to a reduced weighted effect size of left temporoparietal rTMS of 0.44 (95% CI 0.19–0.68), and disappointingly was non-significant at one month’s follow-up. 

### 4.5. Challenges for Connectivity Studies

There are definite trends in altered connectivity between those with schizophrenia, at all stages of illness history, and healthy controls. However there have been inconsistencies in outcomes between studies. Heterogeneous data suggests different mechanisms might be involved in a clinically variable spectrum disorder, with both aberrant local synaptic processes and more macroscopic association fibre miswiring plausible explanations, either alone or in combination. Alterations between those with schizophrenia and healthy controls persist whichever study paradigms, participant criteria and connectivity analyses are used, but analytical methods are challenged for the biases they can introduce and the temporal resolution of fMRI in particular remains relatively poor [88].

Linking macro-level connectivity alterations with pathological cellular and intracellular level changes is incomplete. One could, broadly speaking as part of the neurodevelopmental model of schizophrenia, conceptualise an iterative process of cellular level changes adversely affecting the development of appropriate connection hubs and hierarchical organisation, which could further topographical development and divisional organisation of the brain, further affecting cellular function [129].

Though the existing data show similar patterns of connectivity changes in high/ultra-high risk groups who have not (and may not) develop any illness, there is currently insufficient data to confidently state how closely overall changes and specific symptoms in schizophrenia can be applied to other patient groups and “healthy hallucinators”. Further there is a lack of longitudinal follow-up data to assess how, or if, changes occur with time, and how any such map on to the clinical phenotype and symptoms. This work should also account for the confounder of psychotropic medication that, as well as affecting symptom expression has also been shown to have longer-term effects on brain structure: indeed such work offers the possibility of far better elucidating if such changes are correlated with illness projector. 

Finally, regarding rTMS, once again a highly critical eye must be cast on the generally small sample sizes: as noted in Section 3.5, concerns arise about underpowered studies producing statistically significant but small effect sizes, and the propensity for similarly sized and designed follow-up work “replicating” such findings, but in reality adding to a propensity for publication bias. At best initial enthusiasm of this novel technique’s potential has been largely tempered by more rigorous, and underwhelming, data from larger studies: significant circumspection must be kept regarding its likely longer-term clinical utility.

## 5. Outcomes in Clinical AVH

Research in psychosis has typically supported a general decrease in positive (though not negative and cognitive) symptoms through the lifespan [106,130]. About half of first-episode patients studied will still have positive and/or negative symptoms a decade later [131], and an international 15- and 25-year follow-up study [132] showed reasonably heterogeneous outcomes, fitting with the concept of a spectrum disorder. Interpretation is inevitably confounded by quite differing participating patient illness profiles, premorbid functioning, social functioning [133], socioeconomic factors and so forth that can hinder direct comparison [134], but a recent systematic review and meta-analysis of 50 studies evaluating recovery [135] showed a disappointing median of 13.5% of patients attaining recovery criteria. Prominent AVH early in psychosis have been associated with longer duration of untreated illness [136], and work by ten Velden Hegelstad *et al.* [137] found that the initial presence of hallucinations was the symptom most strongly associated with non-remission at ten-year follow up.

**Figure 3:**
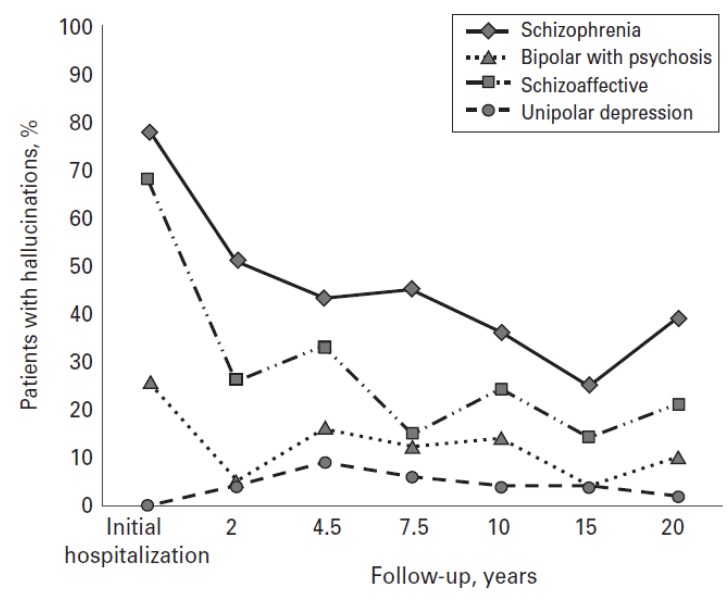
Longitudinal trajectory of hallucinations in patients with schizophrenia, schizoaffective disorder, bipolar disorder with psychosis and unipolar depression over a 20-year period. Adapted with permission from [138].

Prospective longitudinal outcome data focusing on AVH are less common than that on illness recovery more generally [6]. Goghari *et al.* [138] undertook the first prospective long-term follow-up, with 150 patients (schizophrenia 51; schizoaffective disorder 25; bipolar affective disorder with psychosis 25; unipolar depression 49) over twenty years at six different time points. The early presence of AVH predicted poorer outcome and hallucinatory intensity was correlated with reduced employment attainment in all groups. 54% of patients with schizophrenia and 20% of patients with schizoaffective disorders demonstrated either frequent or chronic hallucinations over the full study time-course (Figure 3). The authors reasonably hypothesise their data illustrates both how positive symptoms tend to wane with time (with the caveat that the initial samples were taken during hospitalisation), and also how nevertheless a significant illness burden from AVH remains, particularly in schizophrenia. Whilst negative and cognitive symptoms have generally been regarded as better predictors of subsequent impaired psychosocial attainment this data also demonstrated a relationship between AVH persisting in the initial few years of the study and poorer real-world functioning. 

## 6. Conclusions

Perceiving voices when there is no external stimulus is a more common phenomenon than might be predicted. AVH do not necessarily indicate mental ill-health, and a sense of control over them and the level of distress they cause are key factors in how likely or otherwise individuals are to come to clinical or professional attention. There is growing data in mental health generally for spectrum disorders, with sufferers of varying symptoms having varying degrees of dysfunction across domains: genes and environment are important risk loading factors, and there is a continuum of individuals from those who remain subclinical to those who progress to a diagnosable psychiatric illness. 

Two neurocognitive models have received the most support, the feed-forward model and the aberrant memory model, and there is evidence to support them though both have attracted criticisms and are undoubtedly incomplete. It is possible, and the evidence would appear to indicate it is likely, that there is not a single pathological process that covers all those with AVH. This is further borne out by neuroimaging and connectivity analysis that shows general patterns of cerebral indication strongly indicating an aberrant frontotemporal network involving speech, memory and salience processes but with reasonable variation between studies. The incompleteness of current models is highlighted by how indirectly AVH fit into the predominant pharmacological model of schizophrenia, the dopamine hypothesis.

Connectivity data show brain changes occur premorbidly in subclinical populations with AVH, in those at high risk who do not develop psychotic illnesses and in those who will develop schizophrenia. Inevitably this raises the question of whether or not specific brain changes could act as biomarkers. Currently the sensitivity, specificity and predictive of such changes is highly challengeable, though it is conceivable that along with genetic testing (and of course existing practices such as reviewing family histories, drug use, psychosocial stressors and so forth) that it could contribute to future risk profiling. However it also raises intriguing possibilities for future work to combine genetics and imaging to explore how changes in the former affects the development of brain structure, function and connectivity. 

Longitudinal data on AVH are sparse, particularly in non-psychotic populations, and whilst “positive” symptoms of psychosis such as AVH tend to reduce with time, there is evidence of a significant persisting burden later in life for many. Given the disappointing results of antipsychotic medications for many, and the difficulties engaging some with psychological therapies there is a need to develop novel therapies. rTMS is emerging as both an investigative tool of underlying cortical processes and as a therapeutic agent for AVH though more recent results have tempered initial enthusiasm. Whether or not rTMS will have a more mainstream role in future therapies will be determined by better understanding it mechanisms of action in the brain and the optimal parameters of use.
